# A reinforcement learning approach to explore the role of social expectations in altruistic behavior

**DOI:** 10.1038/s41598-023-28659-0

**Published:** 2023-01-31

**Authors:** Rosendo Castañón, Fco. Alberto Campos, José Villar, Angel Sánchez

**Affiliations:** 1 IdEst Advisory, Madrid, Spain; 2 Instituto de Investigación Tecnológica-Universidad Pontificia Comillas, Madrid, Spain; 3 INESC TEC-Institute for Systems and Computer Engineering, Technology and Science, Porto, Portugal; 4 Grupo Interdisciplinar de Sistemas Complejos (GISC), Departamento de Matemáticas, Universidad Carlos III de Madrid, 28911 Leganés, Spain; 5 Instituto de Biocomputación y Física de Sistemas Complejos (BIFI), Universidad de Zaragoza, 50018 Zaragoza, Spain

**Keywords:** Information theory and computation, Human behaviour, Psychology and behaviour, Applied mathematics

## Abstract

While altruism has been studied from a variety of standpoints, none of them has proven sufficient to explain the richness of nuances detected in experimentally observed altruistic behavior. On the other hand, the recent success of behavioral economics in linking expectation formation to key behaviors in complex societies hints to social expectations having a key role in the emergence of altruism. This paper proposes an agent-based model based upon the Bush–Mosteller reinforcement learning algorithm in which agents, subject to stimuli derived from empirical and normative expectations, update their aspirations (and, consequently, their future cooperative behavior) after playing successive rounds of the Dictator Game. The results of the model are compared with experimental results. Such comparison suggests that a stimuli model based on empirical and normative expectations, such as the one presented in this work, has considerable potential for capturing the cognitive-behavioral processes that shape decision-making in contexts where cooperative behavior is relevant.

## Introduction

Altruism, understood as those actions developed with a deliberate focus on benefiting others, rather than oneself (such as the decision to participate in activities like recycling, blood donation, or programs to support the poor), can play an essential role in shaping the future of modern societies, and studying it could provide insight into the mechanisms that drive it. Ultimately, understanding said mechanisms could serve to inform public and regulatory entities about widespread collaborative behavioral traits, thus equipping them to develop more comprehensive, holistic and effective regulatory frameworks to address some of the most relevant sociological problems, such as the climate emergency^1,2^, the emergence of social inequality^3^ or increasing socioeconomic polarization^4,5^.

Biology, psychology, and classical economics were the first disciplines to study the emergence of altruism, and as such, laid the foundations on which more modern theories are built. In biology, the rise of altruism was never fully understood and was actually quite debated. Charles Darwin himself considered that altruistic behavior was anomalous within the framework of his theory of evolution and that it could even put his theory at risk^6^; in fact, it took several decades to resolve (at least partially) the so-called "altruism paradox" in Darwinian theory, since it was necessary to introduce the notions of inclusive fitness as well as group selection and kinship^7^. In any case, these theories, which propose that natural selection favors the survival of groups over that of individuals as a way of ensuring the legacy of the genetic material to the future generations, seem to be insufficient to provide a conclusive view on the origins of altruism, and presents some controversy and limitations with regards to its applicability^8^.

In the psychological literature of the early twentieth century, theories based on an intrinsically selfish or egocentric motivation of the human being were proposed to explain altruistic behavior^9^, among which the pseudo-altruistic approach was (and still is) particularly dominant^10^. This approach states that the ultimate goal of manifesting altruistic behavior lies in maximizing individual well-being by attaining internal rewards (which may or may not be observable). In a similar vein, at the end of the twentieth century, reciprocal altruism was proposed^11^, based on the idea that collaborative initiatives are only established with the intention of reciprocity in favor of the accepting party. Anyhow, the review of the psychological literature presented in Ref.^10^ concludes that traditional versions of altruism based on narrowly defined selfish tendencies are insufficient to explain the richness of aspects observed in altruistic behavior, insisting that it is not possible to attribute motivations for altruistic behavior by analyzing intrapersonal influences alone, i.e., understanding altruism requires including interpersonal influences. However, alternative frameworks that could incorporate a more holistic vision of self-interest, incorporating other social phenomena such as reputation or social desirability could provide a more integrated and complete framework from which to study prosocial behavior from an egocentric standpoint^12^.

In the arena of sociology and economics, which study interpersonal relationships in large-scale or complex systems, classical theories encountered limitations in their explanation of altruism, essentially due to a tendency to over-rationalize individuals and due to the prevalence of utilitarian theories^13–15^. However, contemporary versions of these disciplines, which tend to integrate non-rational traits on human conduct, propose that expectations about others’ decisions (i.e., social expectations) could be closely linked to the rise of altruistic behavior. This is primarily due to the success of theoretical predictions derived from associating the formation of expectations with consequent behaviors in social contexts proposed in behavioral economics experiments, such as herd behavior^16^, strategic thinking in contexts of bounded rationality^17^ or cooperations in games^18^. In this context, the social norm concept introduced by Bicchieri^19^, that has been proved successful to tackle societally relevant problems^20^, is a strong candidate to help understand the emergence of prosocial behavior^21^. According to Bicchieri, social norms involve (i) empirical expectations (beliefs about what others do), (ii) normative expectations (beliefs about what other people think we should do), and (iii) conditional preferences (understood as the disposition of people to act according to their expectations). In essence, for a social norm to exist, all social expectations (both empirical and normative expectations, which are mediated through empirical and normative interactions, respectively) must be aligned and endorsed by conditional preferences that lead them to act according to these expectations.

Multiple game-theoretical frameworks have been used in behavioral economics to try to capture collaborative behavior and the expression of altruism through expectation formation. Examples are the prisoner's dilemma^22^ (PDG), the public goods game^23^ (PGG), the ultimatum game^24^ (UG), or the dictator game^25^ (DG). Of these, PDG and UG have historically received much attention to analyze cooperation. Still, some studies indicate that in the interaction rules defined by these games, underlying motivations (besides altruism) guide the agents' decisions^26^. In recent years, however, the DG seems to be increasingly recognized as a particularly well-disposed framework for analyzing selfless behavior. The rules of this game are simple: a one-shot game takes place between two players with two different roles, a dictator and a recipient. The dictator receives an amount of money at the beginning of a DG round and is invited to share it with the recipient however she sees fit. In contrast, the recipient passively accepts the distribution made by the dictator without being allowed to make any claim on the received amount. Given that there is no two-way interaction, the DG allows to discard explicit reciprocity as a mechanism to regulate the interactions, and better isolate the effect of other extrinsic and intrinsic incentives, such as acceptability or agreeableness^27^. Experimentally, Engel's meta-analysis^25^ shows that the average donations established in DG experiments, far from being zero, are usually around 30% of the total, with a significant number of agents offering an equitable distribution. Furthermore, some authors point out that the results of the DG could be tightly linked to the social distance between members, with strongly connected players sharing even larger portions of the endowment^28^. In fact, in a recent study, in which the dictator-recipient pairs are configured from couples (in the romantic sense), the average donations reach values close to 60%^29^.

Given the set of interaction rules provided by DG, another fundamental ingredient to understand the emergence of behavioral patterns would be the strategy updating mechanisms that shape agents' future actions. For that matter, experiential induction (commonly referred to as reinforcement learning^30^) is recently showing great results as a method to define evolutionary dynamics (see Refs.^31,32^ for that matter), especially when used to understand collaborative contexts. In particular, one of the theory's successes is the correct prediction of the highly heterogeneous behavior of the population when experiments are performed on large populations, i.e. that the distribution of the adopted collaborative strategies has a non-negligible variance, or, conversely, that there is no dominant strategy in collaborative contexts. In line with the above, another great success of the theory is the robustness of the method, since it is able to correctly predict prosocial behavior in a wide range of interaction rules between agents (namely, similar outputs can be observed when using different game-theoretic approaches).

In view of all this, in this paper we propose that integrating (i) social expectations in a (ii) DG-based game theoretical framework, incorporating (iii) reinforcement learning strategic updating, might be an appropriate pathway to build a model providing insight on the driving forces of altruistic behavior. To support this approach, we will start from Ref.^33^, which presents an agent-based model where interactions are governed by expectations set by DG rules (in a fully connected network), and agents react to empirical stimuli based on the Bush–Mosteller reinforcement learning dynamics^34,35^. The model in Ref.^33^ succeeds in reproducing some of the most important behaviors observed in experiments but at the same time it presents some shortcomings to explain the emergence of altruism as something arising from expectation formation. Some of these limitations are (i) the absence of normative expectations, (ii) the lack of variability and dynamics in the agents characterization (in Ref.^33^, susceptibility to stimuli, i.e. the degree to which stimuli influence future strategies, which could be also understood as a “learning” parameter, is the same for all agents and does not evolve in time), and (iii) the identical weighting of positive and negative interactions (namely, whether the interaction results in an agent enlarging or diminishing her aspiration levels). The main objective of this paper is to propose a new model that addresses these shortcomings by improving the representation of some of the characteristic human behaviours observed in DG settings, namely, (i) that average donations are close to 30% of the initial endowment, (ii) the highly heterogeneous donation profile of the population and (iii) the existence of a non-trivial number of humans that decide against a collaborative approach.

## General model

Let there be *N* agents interacting iteratively through a Dictator Game. At the beginning of each round, agents are arbitrarily split into dictators and recipients, and dictator-recipient pairs are assigned randomly (in a fully connected network^36^). At this stage, prior to the empirical interactions between dictators and their corresponding recipients (in which each dictator decides how to split an initial endowment $\Phi$), normative interactions between dictators take place, with each dictator directing and receiving normative stimuli to and from a number $Q$ of peers. Said normative interactions are modelled by emulating conversations between dictators who share their aspiration levels (i.e., the portion of the endowment they see fit to keep to themselves), with each other (the idea that conversations normatively stimulate individuals is rooted on^37^). Dictators then update their strategy by modifying their aspirations (in other words, strategies are determined by aspirations), taking into account the apparent beliefs of other dictators and being somewhat compliant with them. Following up, dictators give their respective recipients a share of the endowment that is consistent with their updated aspiration, and the empirical interaction occurs. Recipients then experience an empirical stimulus, given by the degree of misalignment between their aspirations (the amount they were expecting to receive) and the quantity perceived, which will then be used to update their future aspirations.

**Figure 1:**
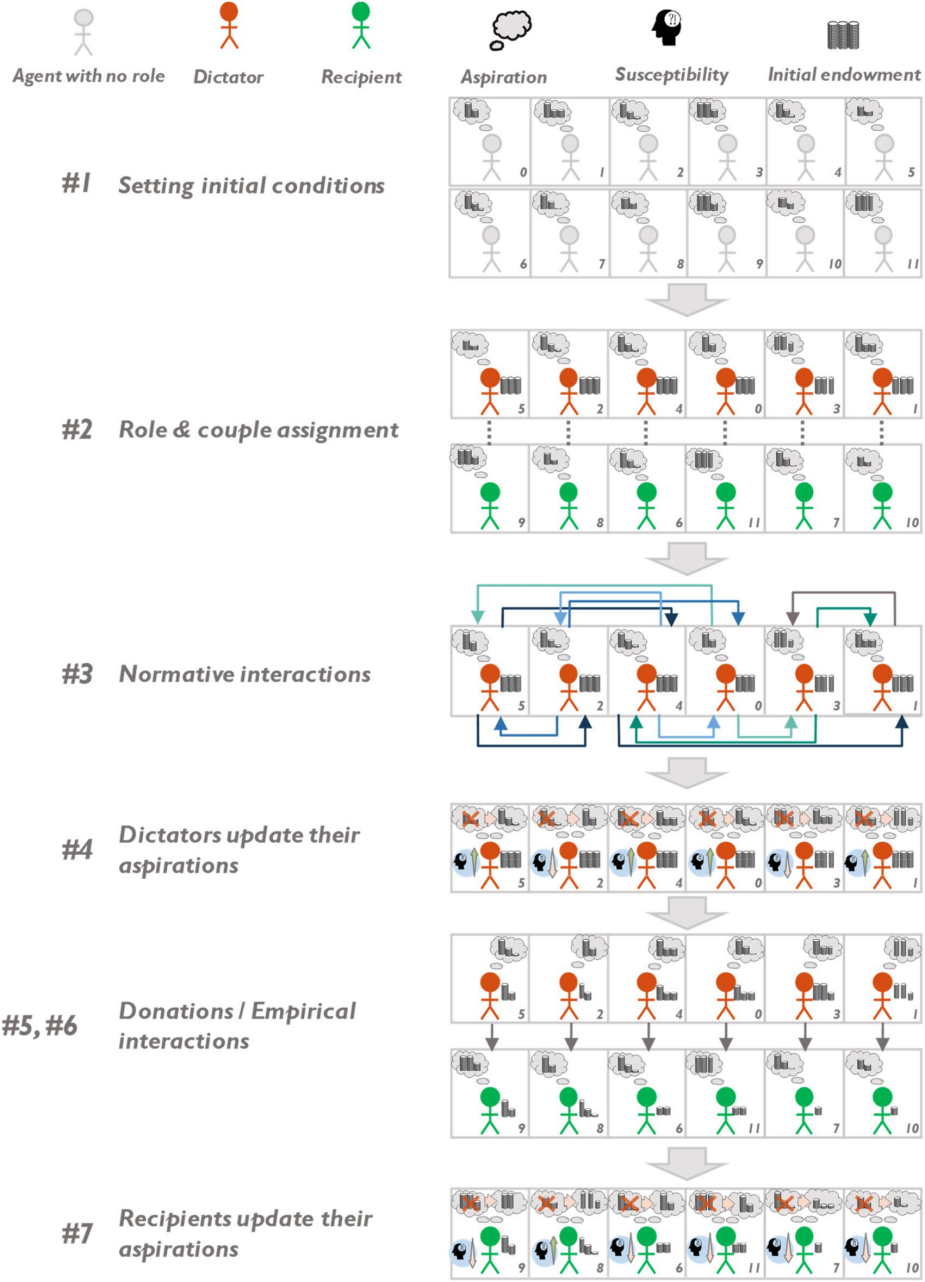
Visual representation of the different phases of the model, for a value $Q$ = 2, shown by the number of arrows coming out and from each dictator in step #3.

As can be seen with this conceptual scheme, in contrast with^33^, where only empirical expectations are modelled, both normative and empirical interactions are mathematically modeled, meaning they both have a role in defining future actions. In the context of this work, only dictators experience potential variations of their aspirations due to normative interactions, while recipients only experience potential variations of their aspirations due to empirical interactions. However, since roles are randomly set for each round, agents will get to incarnate both roles at some point in time, so all agents are exposed to fluctuating normative and empirical expectations. From a more quantitative and algorithmic perspective, the model, which is inspired by Bush–Mosteller algorithm, proceeds as follows (steps correspond with those shown in Fig. 1):

1. *Initial conditions* In $t=0$, agents are endowed with a certain level of initial aspiration, ${a}_{i,t=0}$ in the range [0$,\Phi$] (with $\Phi$ being the dictator’s endowment), which reflects the amount of money they expect to obtain in the first round of DG. Furthermore, agents are given an initial susceptibility level to empirical and normative stimuli$, {l}_{i,t=0}^{emp}$ and ${l}_{i,t=0}^{nor}$. We assume that susceptibility to normative and empirical interactions can be rivalrous, in the sense that growing empirical susceptibility beyond a certain threshold diminishes susceptibility to normative stimuli and vice-versa. These effects can be represented selecting ${l}_{i,t=0}^{emp}$ and ${l}_{i,t=0}^{nor}$ such that:
  1 $$0\le {l}_{i,t}^{emp}+{l}_{i,t}^{nor}\le 1,\mathrm{ with }{l}_{i,t}^{emp}, {l}_{i,t}^{nor}\ge 0.$$

Which will also act as a constraint for any other value of $t>0$ (as explained after Eq. (5)). In this vein, after setting the initial conditions, $t$ is set to 1, to represent the first round of the dictator game taking place.

2. *Role assignments* Agents $(i)$ are assigned into dictator ($j$) and recipient ($k$) roles, randomly, but with equal numbers of dictators and recipients in each round, and random dictator-recipient couples are assigned. For the sake of clarity with regards to index usage, hereinafter, variables and parameters that refer specifically to dictators’ or recipients’ actions/behaviors will use indexes $j$ or $k$, respectively, while index $i$ will serve to describe all agents.
3. *Normative interactions* Dictators honestly communicate their different levels of aspirations to each other. However, possible variations on their state of mind (e.g., temperamental fluctuations, fear of being considered too generous or too greedy) may lead to the value shared by a given dictator $j$, ${\theta }_{j,t}$, to deviate from their actual aspiration level, ${a}_{j,t}$, according to a noise parameter ${\delta }_{j,t}^{nor}$, which intends to account for a "trembling hand" effect, as long as $0\le {\theta }_{j,t}\le\Phi$, i.e.:
  2 $${\theta }_{j,t}=max\left(0,min\left(\Phi ,{a}_{j,t}\cdot \left(1+{\delta }_{j,t}^{nor}\right)\right)\right).$$

Additionally, as a built-in hypothesis for the model, we assume that normative interactions are more frequent than empirical ones. This assumption is in line with^38^ and intends to capture that, when people do not know how to act in a social dilemma (for example, due to lack of experience), they tend to seek for advice from other people, probably more than once, to make moderately informed decisions. In this work we consider that dictators engage in $Q$ normative interactions for every empirical interaction they will later engage in (donating money to their recipient). Every normative interaction ${I}_{j,j{^{\prime}},t}^{nor}$ acts on the focal agent (a specific dictator $j$) through the next term:

3 $${I}_{j,j{^{\prime}},t}^{nor}=\frac{{\theta }_{j{^{\prime}},t}-{a}_{j,t}}{\Phi },$$

where $\Phi$ is used in the denominator $\mathrm{so \; that }-1\le {I}_{j,j{^{\prime}},t}^{nor}\le 1$ (which ultimately will serve to bound the subsequent aspiration levels, ${a}_{j,t}$ in [0$,\Phi$], as will be proved after Eq. (6)). After *Q* such interactions, the normative stimuli ${s}_{j,t}^{nor}$ of dictator $j$ arising from her belief about what other dictators $j{^{\prime}}$ think she should do in a DG situation is given by the average of normative interactions ${I}_{j,j{^{\prime}},t}^{nor}$, i.e.:

4 $${s}_{j,t}^{nor}=\frac{{\sum }_{j{^{\prime}}}{I}_{j,j{^{\prime}},t}^{nor} }{Q}=\frac{{\sum }_{j{^{\prime}}}{\theta }_{j{^{\prime}},t} -{a}_{j,t}{\sum }_{j{^{\prime}}}1 }{\Phi \cdot Q}=\frac{{\sum }_{j{^{\prime}}}{\theta }_{j{^{\prime}},t} }{\Phi \cdot Q}-\frac{{a}_{j,t}}{\Phi }.$$

In other words, we consider that normative stimuli arise from the average difference between the beliefs shared by external players of what they convey they would do, ${\theta }_{j{^{\prime}},t}$ and the player's own aspiration level, ${a}_{j,t}$, which captures the player's intention of action. It is also interesting to note that the stimuli derived from normative interactions are directed, in the sense that it is only the dictator who seeks advice from his peers that updates his expectations. That is, the $Q$ dictators who are consulted by dictator $i$ do not experience a normative stimulus from doing so. Furthermore, note that $-1\le { s}_{j,t}^{nor}\le 1$.

4. *Dictators update their aspirations* The impact of normative stimuli on the aspirations of a dictator $j$ is twofold and has sequential order. Firstly, normative stimuli affect the normative susceptibility ${l}_{j,t}^{nor}$ according to:
  5 $$ \begin{aligned} & l_{j,t}^{nor} = l_{j,t - 1}^{nor} \cdot\left( {1 + W_{ }^{nor,pos} \cdots_{j,t}^{nor} } \right), {\text{if}} s_{j,t}^{nor} \ge 0 \\ & l_{j,t}^{nor} = l_{j,t - 1}^{nor} \cdot\left( {1 + W_{ }^{nor,neg} \cdots_{j,t}^{nor} } \right),{\text{ if}} s_{j,t}^{nor} < 0 \\ \end{aligned} $$
  where ${W}^{nor,pos}$ and ${W}^{nor,neg}$ are input parameters in [0,1] that determine the impact of the normative stimuli on the dictator’s normative susceptibility. Meanwhile, since dictators do not experience an empirical stimulus, their empirical susceptibilities remain unchanged in the round (${l}_{j,t}^{emp}={l}_{j,t-1}^{emp}$). A consequence of this is that, after updating the susceptibility levels of a dictator, ${l}_{j,t}^{nor}+{l}_{j,t}^{emp}$ could be larger than 1. Whenever this happens, in accordance with Eq. (1), ${l}_{j,t}^{emp}$ and ${l}_{j,t}^{nor}$ are recalculated so that ${l}_{j,t}^{nor}{+l}_{j,t}^{emp}=1$, with $0\le {l}_{i,t}^{emp}, {l}_{i,t}^{nor}\le 1,$ and in a way such that they maintain their relative size.

Secondly, normative stimuli $${s}_{j,t}^{nor}$$ influence aspiration levels explicitly (due to the explicit term of $${s}_{j,t}^{nor}$$) and implicitly (due to having modified the susceptibility value $${l}_{j,t}^{nor})$$ as follows:

6 $$ \begin{aligned} & a_{j,t} = a_{j,t - 1} + \left( {{\Phi } - a_{j,t - 1} } \right)\cdot l_{j,t}^{nor} \cdots_{j,t}^{nor} ,\quad {\text{if}}\,s_{j,t}^{nor} \ge 0 \\ & a_{j,t} = a_{j,t - 1} + a_{j,t - 1} \cdot l_{j,t}^{nor} \cdots_{j,t}^{nor} ,\quad {\text{if}}\,s_{j,t}^{nor} < 0. \\ \end{aligned} $$

Since ${a}_{j,t-1}$, ${s}_{i,t}^{nor}$ and ${l}_{i,t}^{nor}$ are bounded to [$0,\Phi$], $\left[-\mathrm{1,1}\right]$ and [0,1], respectively, it follows that ${a}_{i,t}$ $\mathrm{is naturally bounded to the range} \left[0,\Phi \right]$, as previously claimed.

Note that with the evolution rule described in Eq. (5) the agent becomes more susceptible to interactions when exposed to positive stimuli that will enlarge their future aspirations: indeed, from the first expression in Eq. (5) it can be seen that positive stimuli (${s}_{j,t}^{nor}\ge 0$) increase agent’s susceptibility and sequentially, its aspirations (as can be noted from the first expression in Eq. (6)). Conversely, the agent becomes less susceptible to interactions and decreases its aspirations when exposed to negative stimuli, as shown by the second expressions of Eqs. (5) and (6). Conceptually, this adaptation could be framed as “self-serving” since it models agents seeking to acquire/maintain larger levels of aspiration by updating their susceptibility to positive/negative interactions.

In terms of interpreting the equations for a given dictator $j$, positive interactions (${s}_{j,t}^{nor}\ge 0)$ would imply that the average aspiration of other dictators (given by the average of ${\theta }_{j{^{\prime}},t}$, as seen in Eqs. (2) and (4) ) is larger than its own $({a}_{j,t}$), subsequently driving the dictator to augment her personal aspirations (${a}_{j,t}\ge {a}_{j,t-1} )$. The opposite is also true, since negative interactions will drive the dictator to consider that she should lower her aspirations in the future (if ${s}_{j,t}^{nor}<0$, then ${a}_{j,t}\le {a}_{j,t-1} )$.

5. *Dictators proceed to donate* Donations from dictator $j$ in a round$,$ ${d}_{j,t}$, are given by an amount that would be consistent with her level of aspiration ${a}_{j,t}$ (an internally consistent rational agent would never donate more than her own aspiration level), multiplied by a noise parameter, ${\delta }_{j,t}^{emp},$ which models a trembling hand effect for interactions of an empirical nature. This effect is captured through the following equation, where ${d}_{j,t}$ is always adjusted to verify ${0\le d}_{j,t}\le\Phi$, i.e.:
  7 $${d}_{j,t}=max\left(0,min\left(\Phi ,\left(\Phi -{a}_{j,t}\right)\cdot \left(1+{\delta }_{j,t}^{emp}\right)\right)\right).$$

It is relevant to point out that the amount ${d}_{j,t}$ donated by a dictator $j$ to a recipient $k$ in a game round $t$ becomes the donation ${\pi }_{k,t}$ received by the recipient. Therefore $0\le {d}_{j,t}={\pi }_{k,t}\le\Phi$.

6. Empirical interactions take place for recipients, as they perceive their assigned donation. As a result of a dictator's donation decision, their corresponding recipient receives an empirical stimulus ${s}_{k,t}^{emp}$, which is obtained from the difference between the amount ${\pi }_{k,t}$ received by the recipient $k$ and her level of aspiration ${a}_{k,t}$ in a round $t$.
  8 $${s}_{k,t}^{emp}=\frac{{\pi }_{k,t}-{a}_{k,t}}{\Phi }.$$

This equation represents that, whenever there is a misalignment between the aspiration level ${a}_{k,t}$ and the amount perceived ${\pi }_{k,t}$, the recipient $k$ experiences a negative empirical interaction in case ${\pi }_{k,t}<{a}_{k,t}$, or a positive one in case ${\pi }_{k,t}>{a}_{k,t}$, with ${s}_{k,t}^{emp}\in \left[-\mathrm{1,1}\right]$, analogously to ${s}_{i,t}^{nor}$.

7. *Recipients update their aspirations* This process is completely analogous to how dictators updated their aspirations (given by Eqs. (5) and (6)). In that vein, the impact of empirical stimuli on the aspirations of a recipient $k$ is twofold and has sequential order. Firstly, empirical stimuli affect the empirical susceptibility ${l}_{k,t}^{emp}$ of recipients according to:
  9 $$ \begin{aligned} & l_{k,t}^{emp} = l_{k,t - 1}^{emp} \cdot\left( {1 + W_{ }^{emp,pos} \cdots_{k,t}^{emp} } \right), \quad if s_{k,t}^{emp} \ge 0 \\ & l_{k,t}^{emp} = l_{k,t - 1}^{emp} \cdot\left( {1 + W_{ }^{emp,neg} \cdots_{k,t}^{emp} } \right),\quad if s_{k,t}^{emp} < 0, \\ \end{aligned} $$
  where ${W}^{emp,pos}$ and ${W}^{emp,neg}$ are inputs in [0, 1] that represent the empirical component of the "impact levels”, which, as for normative susceptibilities, indicate the magnitude of the effect of the stimuli on the susceptibility. As was the case with dictators, recipient’s susceptibilities can be set to satisfy Eq. (1), and therefore $0\le {l}_{k,t}^{emp}\le 1$.

Secondly, empirical stimuli influence aspiration levels explicitly (due to the explicit term of $${s}_{k,t}^{emp}$$) and implicitly (due to having modified the susceptibility value $${l}_{k,t}^{emp})$$ in the following equation, which is again consistent with the " self-serving " adaptation previously described:

10 $$ \begin{aligned} & a_{k,t} = a_{k,t - 1} + \left( {{\Phi } - a_{k,t - 1} } \right)\cdot l_{k,t}^{emp} \cdots_{k,t}^{emp} , \quad if\,s_{k,t}^{emp} \ge 0 \\ & a_{k,t} = a_{k,t - 1} + a_{k,t - 1} \cdot l_{k,t}^{emp} \cdots_{k,t}^{emp} ,\quad if\,s_{k,t}^{emp} < 0, \\ \end{aligned} $$

where, again, carrying the analogous principles shown when describing normative interactions, it follows that ${a}_{k,t}$ $\in [0,\Phi ]$.

8. *New iteration* Completing steps 1–7 for the first time translates into finishing the first round of the DG. From here on, agents are taken back to step 2 and a new round begins so that $t=t+1$, with agents carrying their aspirations from previous rounds, instead of re-initializing them, but with roles being randomized for the upcoming round.

## Simulations

The model presented above is allows to address many aspects of the behavior observed in the Dictator game. However, in order to get more insight into its significance and, above all, into the effects of the different parameters, we now focus on a specific case described below.

### Specific assumptions

I. We assume that agents have a certain predisposition to selfishness. In this vein, initial aspiration ${a}_{i,t=0}$ are drawn from a uniform distribution that is in the range $\left[\frac{1}{2}\Phi ,\Phi \right]$.
II. The initial susceptibility to empirical expectations ${l}_{i,t=0}^{emp}$ is drawn from a uniform distribution in the interval $\left[0, 0.5\right]$, and the initial susceptibility to normative expectations is set as ${l}_{i,t=0}^{nor}$ $=0.5-{l}_{i,t=0}^{emp}$.
III. We will assume that agents hold a unique susceptibility parameter, namely ${l}_{i,t},$ to account for both their empirical and normative interactions (i.e., ${l}_{i,t}^{nor}={l}_{i,t}^{emp}={l}_{i,t}$). Conceptually, this would imply that agents would be "socially consistent" in the sense of being equally reactive to empirical and normative stimuli of the same magnitude. We justify in the annex why the “socially consistent” version of the model is relevant (compared to a more general case where ${l}_{i,t}^{nor}$ and ${l}_{i,t}^{emp}$ do not have to be necessarily equal) by referring it to the corresponding experimental results, as will be furtherly explored in “Discussion”.
IV. As shown in Eq. (4), normative interactions are assumed to be more frequent than empirical ones. In particular, we consider that for every empirical interaction (dictator–recipient interaction), $Q$ normative interactions (dictator–dictator interaction) take place. In the following, we assume $Q=2$, following the assumption of Ref.^38^ that considers a ratio of 2:1 for normative-to-empirical interactions (more specifically for each ingroup and outgroup interactions). We also leverage the considerable frequency of humans to interact normatively with their two parents in the first stages of their learning process. However, it is to be noted that varying this parameter, for instance, in the range {2,…,5}, does not alter the results obtained from the simulations in a significant way, since the shape and mean of the donation and susceptibility distributions remained essentially unchanged.
V. Regarding trembling hand effects, humans are assumed to be more susceptible to conduct themselves in a way that is inconsistent with their beliefs in empirical settings than in normative ones (where there are no incentives to be dishonest). In this vein, the variables that account for the trembling hand effect, ${\delta }_{j,t}^{nor}$ and ${\delta }_{j,t}^{emp}$, are drawn respectively, from gaussian distributions $N(\mathrm{0,0.05})$ and $N(\mathrm{0,0.1})$, implying that fluctuations do not have a systematic positive or negative impact (both distributions have zero mean), and that temperamental fluctuations in empirical interactions can potentially be more pronounced than those occurring in normative interactions (since the former has a larger variance).

### Input scenarios

In the case studies, we explore the parameter space that defines the susceptibility evolution rules through the parameters ${W}^{nor,neg}, {W}^{emp,neg}, {W}^{nor,pos} \mathrm{and }{W}^{emp,pos}$ of Eqs. (5) and (9), discretizing the spaces with values for each of the coefficients, in intervals of 1/3, from 0 to 1. However, for the sake of simplicity in the discussion of the results, only the most representative results are presented (note that there are ${4}^{4}=256$ results for each of the case studies performed).

Each combination of the model parameters (referring to the impact levels) has been replicated 10 times selecting different seeds to generate the outcomes of the probability distributions, and the results averaged. The standard deviations of the averaged donation output distributions are only shown in those cases where they are relevant. i.e., wherever the standard deviation across average donations is larger than 5% of the mean average donation.

### Results

The simulations performed on the DG model described in the previous section consist of 1000 players interacting over a 100-round transient to reach a convergence or permanent regime for the average aspiration level of the agents (defined by a threshold change in the average aspiration level of the agents lower to 0.01%). If a permanent regime is not achieved, the system is allowed to progress in batches of 10 rounds until convergence is reached, however, for the specific case studies developed, a permanent regime is always obtained in the first 100-round transient.

According to assumptions II and III, we initialize ${l}_{i,t=0}$ from a uniform distribution in the interval $\left[0, 0.5\right]$. We begin the presentation of our results setting ${W}^{emp}=0$ but allowing ${W}^{nor}$ to vary. Afterwards, we present the parallel case, with the normative level ${W}^{nor}$ equal to 0 and the empirical impact level varying*.* In all cases, histograms are used to represent the profiles of donations and susceptibility levels of the agent population for each corresponding combination of the varying impact levels (${W}^{nor,neg}$ and ${W}^{nor,pos}$ or ${W}^{emp,neg}$ and ${W}^{emp,pos}$, depending on the case), once the steady state solution has been reached. Note that when this state is reached, the donation’s histogram presents the last donation produced by each agent on its last round as a dictator, moreover, a dashed vertical line is used to visually convey the average value of donations once the steady state has been reached. With regards to the representation chosen for susceptibility profiles, histograms have been considered in the main body of the text, where results are one-dimensional in nature (since ${l}_{i,t}^{emp}={l}_{i,t}^{nor}={l}_{i,t}$ in this case-study, as expressed in assumption I), while scatterplots have been used in the annex (where results are two-dimensional, considering that ${l}_{i,t}^{nor}\mathrm{and }{l}_{i,t}^{emp}$ can have different values in the case study thereby presented). Furthermore, box plot diagrams have been incorporated to each figure to convey a simplified outlook of the histograms and facilitate their comparisons, to some extent.

**Figure 2:**
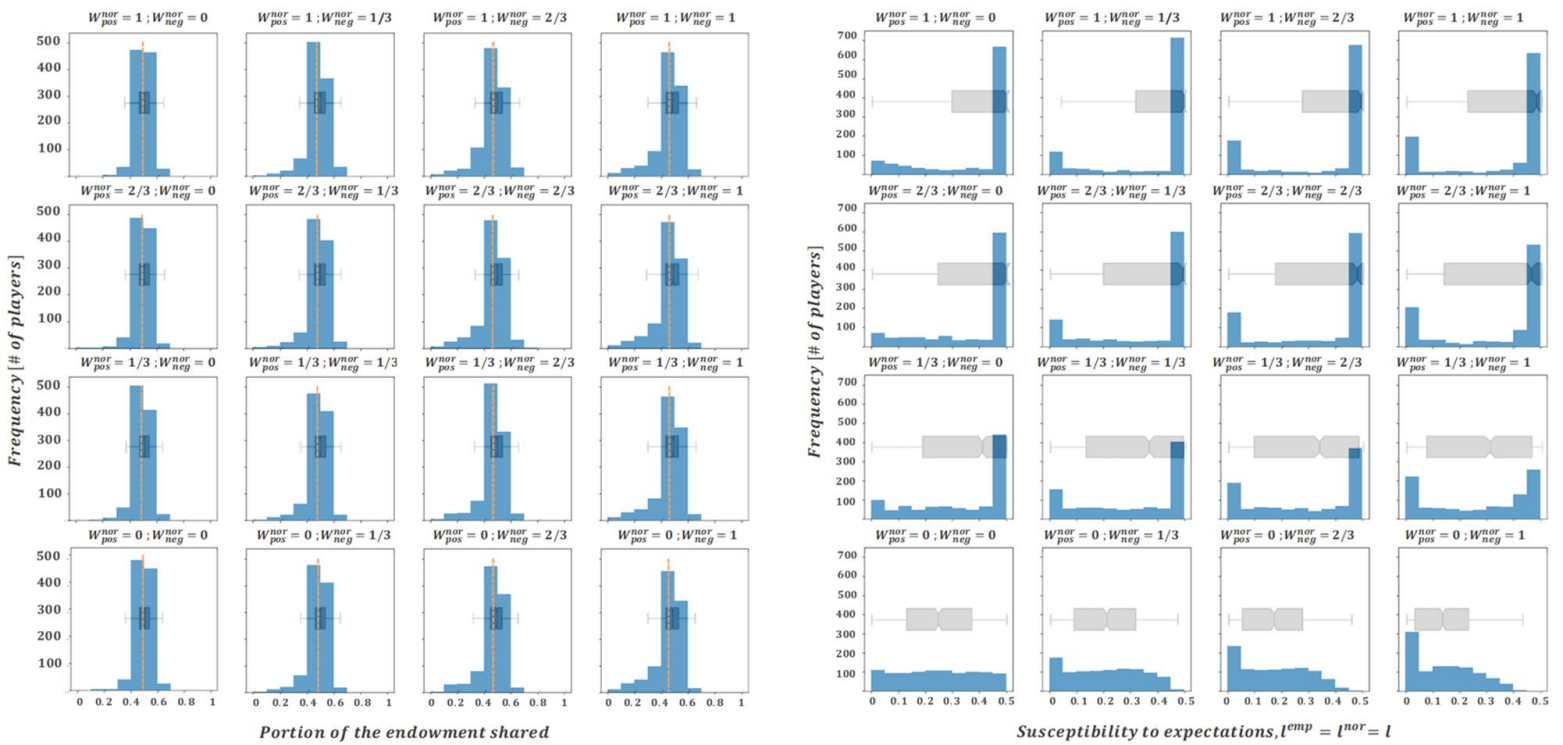
Final averaged distributions of donations (left) and susceptibility profiles with ${l}_{i,t}^{nor}={l}_{i,t}^{emp}$(right) for ${W}^{emp}=0$. Each subplot represents results for the scenarios of ${W}^{nor,pos} \left(\mathrm{which varies across rows}\right)\mathrm{ and }{W}^{nor,neg} \left(\mathrm{which varies across columns}\right)$ assumed in *B*)*.*

**Figure 3:**
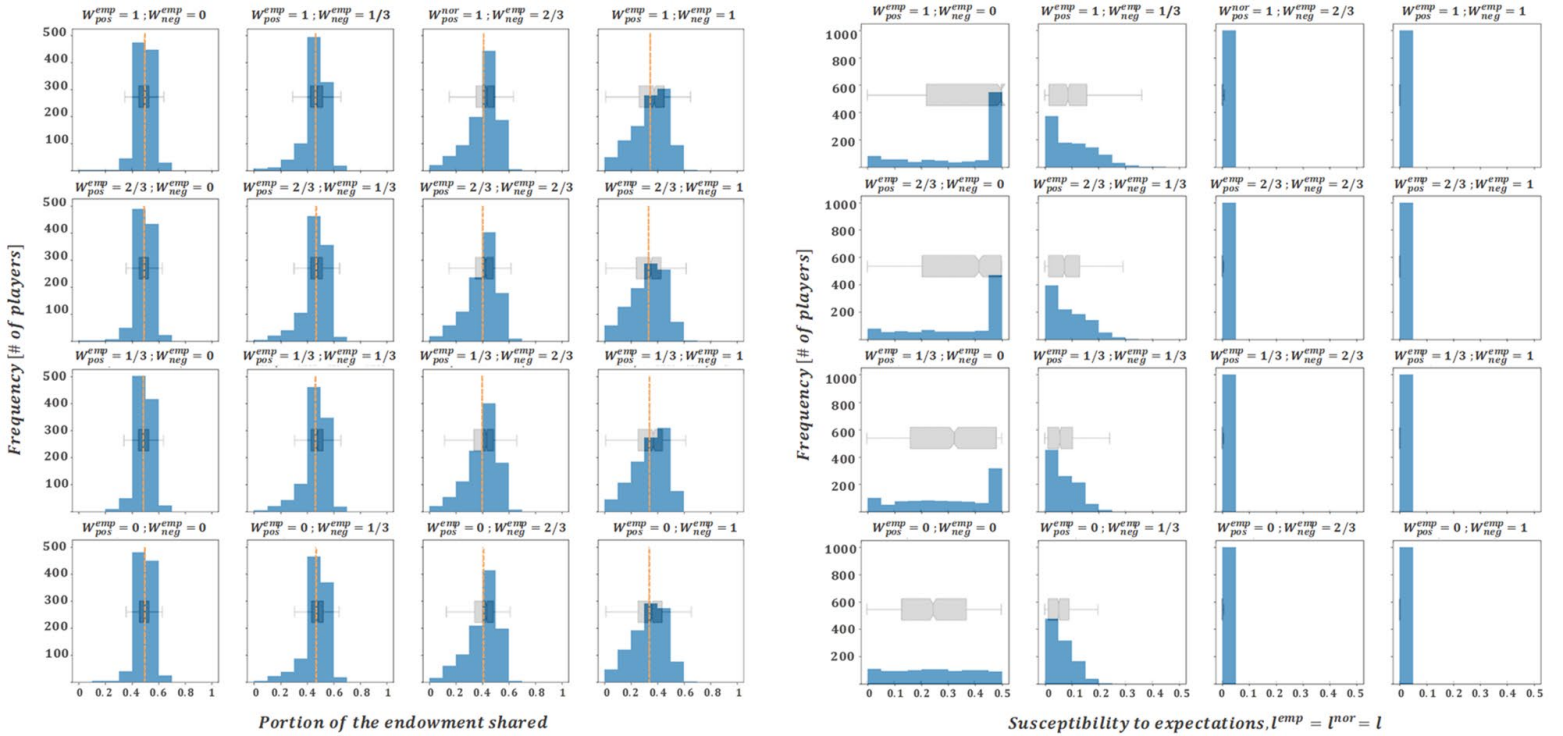
Final averaged distributions of donations (left) and susceptibility profiles with ${l}_{i,t}^{nor}={l}_{i,t}^{emp}$(right) for ${W}^{nor}=0$. Each subplot represents results for a given combination of ${W}^{emp,pos} \left(\mathrm{which varies across rows}\right)\mathrm{ and }{W}^{emp,neg} \left(\mathrm{which varies across columns}\right)\mathrm{ assumed in B})$.

We begin by noting that the subplots presented in the lower left corner of Figs. 2 and 3 ($${W}^{nor,pos}={W}^{nor,neg}={W}^{emp,pos}={W}^{emp,neg}=0)$$ show the final state of the system with the initial susceptibility levels kept constant in time since $${l}_{i,t}={l}_{i,t=0}$$ holds true for any round $$t$$ and any agent $$i$$, as can be easily inferred from Eqs. (5) and (9). It can then be observed that in this case the outcome is an equitable sharing solution in the stationary regime, as the average grant is located close to the 50% of the endowment.

When agent susceptibility is allowed to evolve, the results suggest that negative interactions seem to govern agents’ dynamics. This follows from the fact that changes across rows (over ${W}^{nor,neg}$ in case of Fig. 2 or ${W}^{emp,neg}$ in case of Fig. 3) transform the distributions of donations (and the evolution of susceptibilities) in a more significant way, compared to the changes across columns. Specifically, negative empirical interactions seem to be especially dominant, since variations in ${W}^{emp,neg}$ induce a larger change on both donation profiles and susceptibilities, when compared to the influence of ${W}^{nor,neg}$. One of the possible explanatory components of this effect (empirical interactions governing normative ones) is that normative interactions are calculated from the averaged result of a number $Q$ of dictators' interactions and would thus smooth extreme stimuli, while empirical interactions are one-shot, and so, potentially more extreme. Furthermore, varying $Q$ in the range^2,5^ does not change the results significantly. This means that the designed model, given the outlined hypotheses, favours empirical stimuli to be larger and, hence, more relevant than normative stimuli in shaping future decisions, which is furtherly explored in section “Discussion”.

In line with the previous observation, it seems that increasing the values of ${W}^{nor,neg}$ and (especially) ${W}^{emp,neg}$ drives the average of donations closer to the value proposed in Engel's meta-analysis (average donations are circa 30% of the initial endowment). Interestingly, when ${W}^{emp,neg}\ge 2/3$, the susceptibility profiles tend to cluster around the null value (see the right columns of Fig. 3), suggesting that in these cases being susceptible to stimuli is not necessary to maintain the steady-state behaviour.

**Figure 4:**
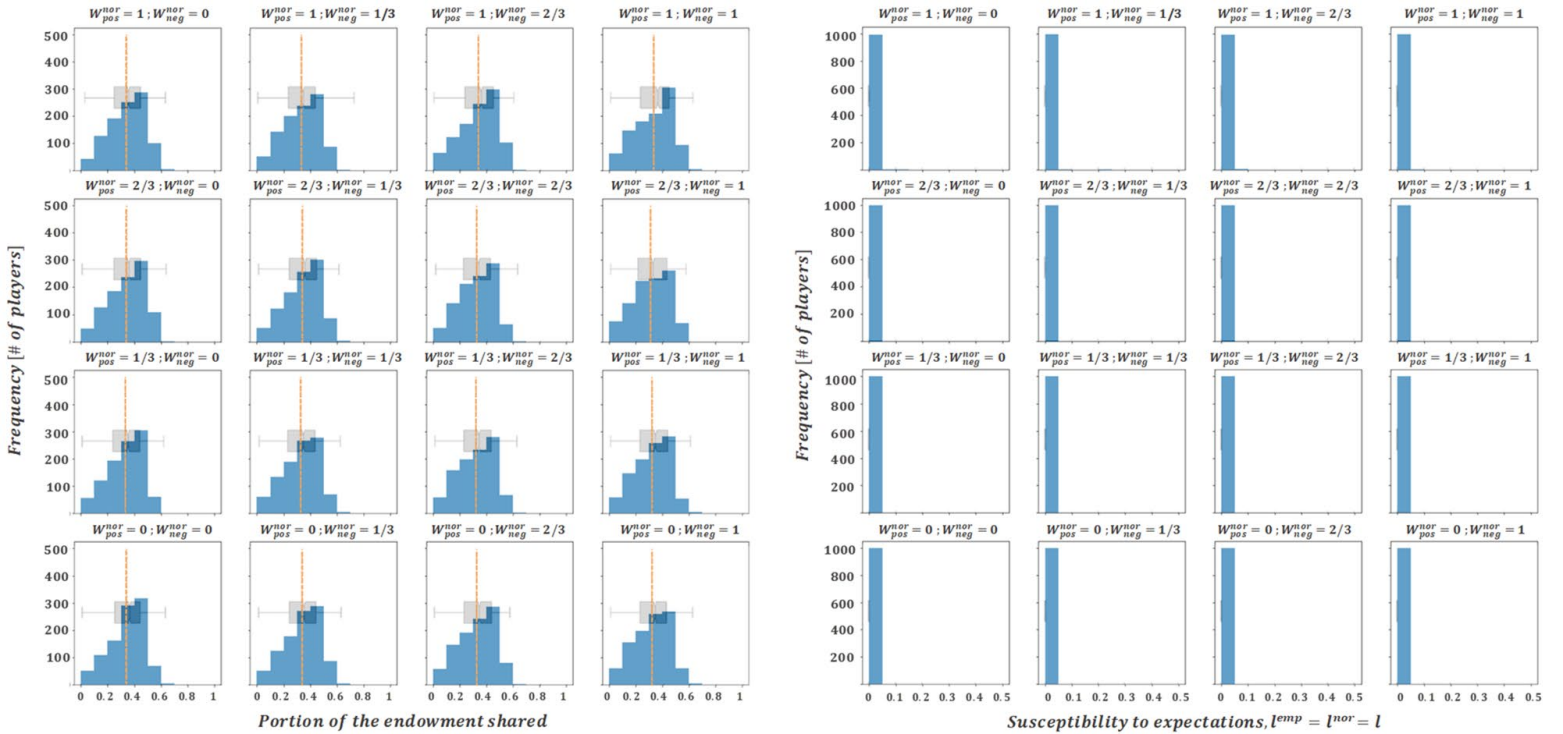
Final averaged distributions of donations (left) and susceptibility profiles with ${l}_{i,t}^{nor}={l}_{i,t}^{emp}$(right) for ${W}^{emp}=1$. Each subplot represents results for the scenarios of ${W}^{nor,pos} \left(\mathrm{which varies across rows}\right)\mathrm{ and }{W}^{nor,neg} \left(\mathrm{which varies across columns}\right)$ assumed in *B*).

**Figure 5:**
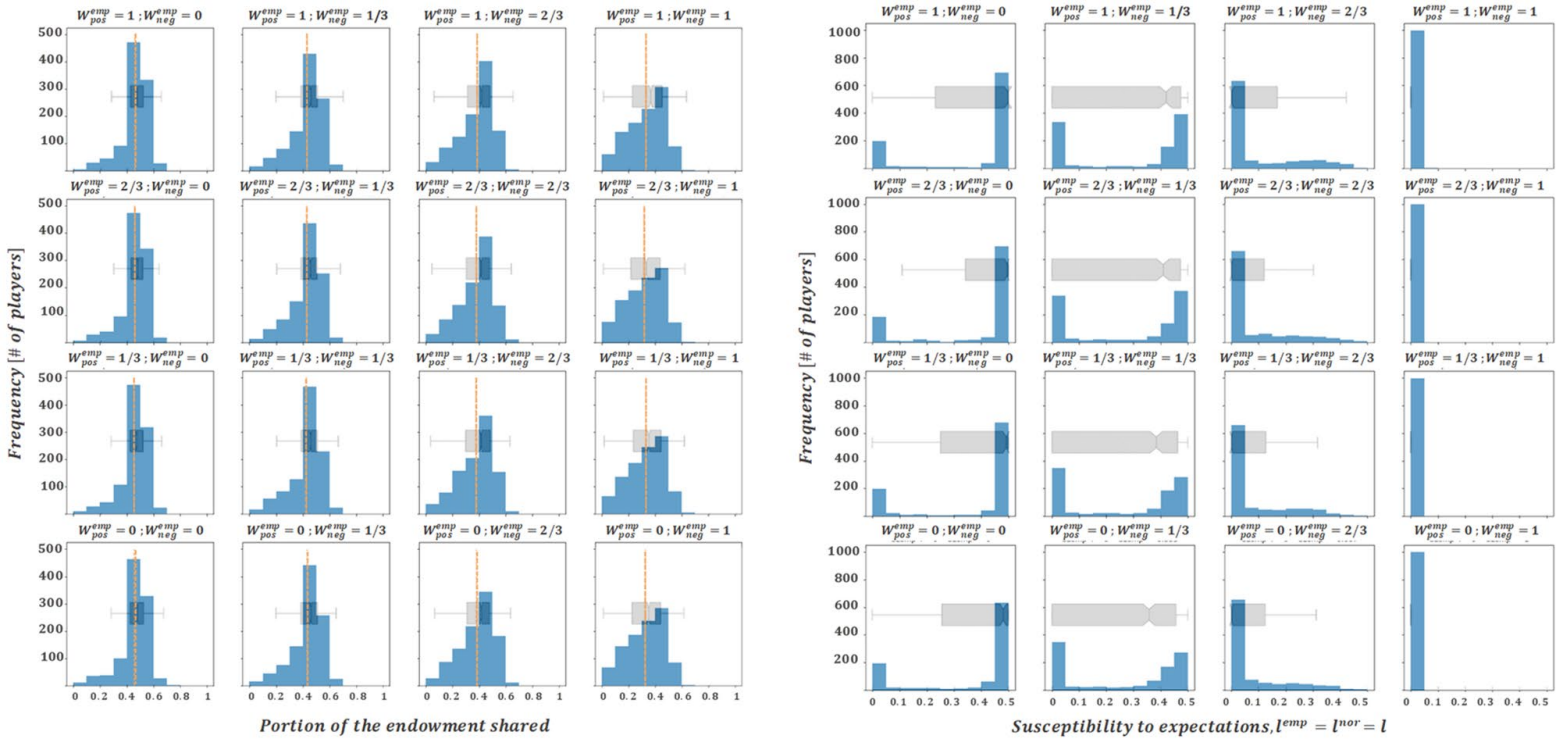
Final averaged distributions of donations and susceptibility profiles for ${W}^{nor}=1$ and ${l}_{i,t}^{nor}={l}_{i,t}^{emp}$. Each subplot represents results for a given combination of the remaining impact levels, ${W}^{emp,pos}\left(\mathrm{which varies across rows}\right) \mathrm{and }{W}^{emp,neg} \left(\mathrm{which varies across columns}\right)$.

Having studied a scenario where ${W}_{ }^{emp}=0$ and another one where ${W}_{ }^{nor}=0$, and given that average donation profiles have shown to be closer to those collected in the literature when the *W* parameters are greater (especially for those accounting for negative interactions), we deem it interesting to study the cases when ${W}_{ }^{emp}=1$ and ${W}_{ }^{nor}=1$. The results for this case are collected in Figs. 4 and 5.

At a glance, Figs. 4 and 5 support the two most relevant insights derived from the previous set of results (Figs. 2 and 3), i.e. that empirical interactions are more relevant than normative ones in shaping the final donation profile, and that the weight of negative interactions (especially of empirical nature) is particularly explicative of the equilibrium solution. In addition, it seems that the larger ${W}_{ }^{emp}$, the lower the relevance of ${W}_{ }^{nor}$ in shaping the final donations profile. This can be seen in the donation subplots presented in Fig. 4 (${W}_{ }^{emp}=1$), which show a greater resemblance between themselves than the subplots in Fig. 2 (${W}_{ }^{emp}=0$). Moreover, it should be noted that all of the donation profiles presented in Fig. 4 are strongly aligned with experimental results presented in Ref.^39^. Indeed, the average donations are in the [30%, 40%] range of the endowment, the modal decision is close to the equitable (50–50) division, and the donations are highly heterogeneous, with a non-trivial portion of agents offering (close to) zero amounts and almost no agent offering more than half of the initial endowment. This is an important result in so far as it gives support to our choice of coupling empirical and normative susceptibilities (motivating assumption IV) When we relax this assumption the results show a large loss of heterogeneity in the final donation profiles, in strong disagreement with the experiments (see the Annex).

Figures 3 and 5 convey a similar message in the sense that the weight of negative empirical interactions,$${W}^{emp,neg}$$, seems to be determinant in the shaping of the donation profile, while the weight of positive empirical interactions, $${W}^{emp,pos}$$, seems to have an almost negligible effect. However, with regards to the susceptibility profiles, both figures show a relevant difference. While Fig. 3 shows susceptibility levels concentrating in the lower range for $${W}^{emp,neg}\ge 2/3$$, Fig. 5 shows considerably longer-tailed susceptibility distributions for $${W}^{emp,neg}=2/3$$, and only for $${W}^{emp,neg}=1$$, do susceptibility levels cluster in the lower range. This is somewhat significant, in the sense that it shows that large and positive normative impact levels (for instance, $${W}_{ }^{nor,pos}=1)$$ help to conserve higher susceptibility levels up to the final rounds of the game. On the other hand, donation profiles that tend to mirror better the experimental results (such as those shown in Fig. 4, or in the fourth column of Fig. 5), consistently present a negligible susceptibility in the final rounds. This suggests that adult humans (emulated by agents in their last rounds) are no longer susceptible to changes in their altruistic conduct.

## Discussion

In order to discuss the validity of our model, let us summarize first the key experimental results shown in Refs.^25,39^, that point to the fact that the donation profile of agents interacting according to DG rules is essentially characterized by three factors:

1. The average donation is close to 30% of the initial endowment to be distributed.
2. The distribution of donations is highly heterogeneous, and, although there are rarely donations above the equitable donation (50–50), the modal decision is close to said equitable donation.
3. There is a non-trivial number of agents (usually around 10% of the total) who decide to take the entire initial endowment for themselves, i.e., they opt for the non-collaborative decision. Furthermore, there tends to be a larger share of agents choosing the non-collaborative decision over the decision of sharing more than 50% of the endowment.

From the results obtained, it is clear that our approach, based on “socially consistent” agents (i.e., agents that share a single susceptibility parameter to account for the effect of normative and empirical interactions on susceptibilities), is very successful in capturing the main experimental findings of^25,39^. Furthermore, the model results show two additional elements that are particularly well aligned with relevant literature on cognitive-behavioral processes.

Firstly, the observation that the overall behavior of the agents is primarily governed by empirical interactions (see the analysis on Figs. 4 and 5), is supported by the work of Bicchieri^20^, which establishes that empirical expectations tend to dominate normative ones, such that beliefs about what others do are much more important than beliefs about what others think one should do in shaping decision making.

Secondly, the fact that negative interactions seem to exert a larger influence on the system compared to positive interactions, is largely backed-up by psychological literature^40,41^. This is also supported by the relevant behavioural economics literature on prospect theory^42,43^, that delves on a fundamental asymmetry of people’s evaluation of utility in settings under risk, with losses being much more relevant than gains. In particular, as can be seen in Figs. 2 and 3, larger weights on negative interactions seem to increase the heterogeneity of the steady-state donation profile and to decrease the average of the donations, leading to results that are closer to those proposed in the DG experimental literature. For that matter, we present a final set of results where the impact levels of negative interactions are set to their maximum levels (${W}^{emp,neg}={W}^{nor,neg}=1$), while the impact levels that influence positive interactions are subject to 1/3 step variations.

**Figure 6:**
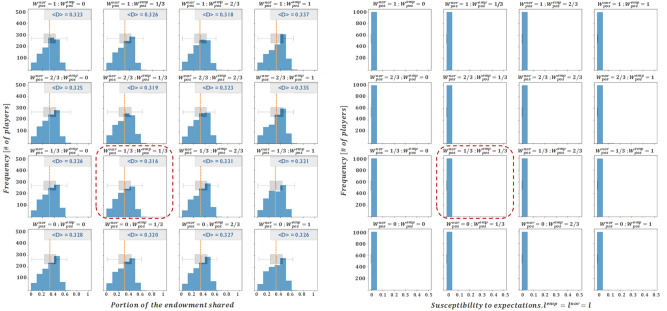
Final averaged distributions of donations and susceptibility profiles for ${W}^{emp,neg}={W}^{nor,neg}=1$ and ${l}_{i,t}^{nor}={l}_{i,t}^{emp}$. Each subplot represents a given combination of ${W}^{nor,pos}$ and ${W}^{emp,pos}$ (which vary across rows and columns, respectively).

While the donation profiles captured in Fig. 6 show a large similarity to each other, and meet most of the criteria outlined at the beginning of this section, the combination of parameters ${W}^{emp,neg}={W}^{nor,neg}=1$, ${W}^{emp,pos}={W}^{nor,pos}=\frac{1}{3}$ is particularly interesting, since it yields the average donation value most closely matched to the literature values (31.6% of the model vs. 30% of the literature) and it is one of the few where the share of selfish behavior is larger than that of dictators that donate equal to or higher than 50% of the endowment.

It therefore seems that a combination of parameters that offers a very high degree of similarity between the results derived from our proposed mathematical/theoretical and on field/experimental field DG results is the one that weights negative interactions three times more than positive interactions (${W}^{nor,neg}=3\cdot {W}^{nor,pos}, {W}^{emp,neg}=3\cdot {W}^{emp,pos}$). Curiously enough, this phenomenon is fully consistent with the psychological literature, which states that even when stimuli are of the same magnitude, positive and negative interactions do not generate symmetrical effects. In fact, experimental evidence suggests that negative dynamics have an impact between 3 and 5 times greater than positive dynamics^44,45^, so that the results of the model with this particular combination of parameters are fully aligned with real results, and signals that distinguishing between positive and negative interactions is relevant in the context of normative and empirical expectations.

Regarding this last scenario, the fact that in the stationary regime, agents' susceptibility to normative and empirical stimuli converges to zero, is indicative that altruistic behavior in mature systems may not require from a social norm to be maintained (recall that for a social norm to exist (see Introduction), social expectations must be endorsed by conditional preferences that lead them to act according to these expectations. In this sense, conditional preferences can be interpreted as the susceptibility parameter used for the model ${l}_{i,t}$, and since the latter variable converges to zero, one can say that there are no conditional preferences present in the stationary regime). That is, mature agents are able to maintain prosocial behavior without being susceptible to normative or empirical stimuli that redirect their behavior in the long run (however, it is essential to emphasize that the fact that agents are not influenced by a social norm in the stationary regime does not imply that they have not been affected by social norms throughout their learning process, especially during the first round of the game, in which susceptibility values are higher). As a consequence, the role of susceptibility in driving collaboration during the initial and intermediate rounds of the game becomes increasingly relevant. In this line, the evolution of agents’ average susceptibility, for the case where ${W}_{ }^{emp,neg}={W}_{ }^{nor,neg}=1$ and ${W}_{ }^{emp,pos}={W}_{ }^{nor,pos}=\frac{1}{3}$ is now presented. Regarding the rate to which the average susceptibility converges to zero as a function of time t, we also present the equation that results from an exponential regression, for illustrative purposes.

Finally, to complete the discussion of the results proposed in this section, it is enriching to present a brief comparison with other important studies that have approached the study of altruistic behavior from a theorical perspective (by proposing a mathematical formulation to capture agent dynamics in collaborative context). In that sense, two works of great interest for this exercise are^46,47^.

Specifically, in Ref.^46^ the authors look at the dictator game from a collectivist point of view (so that its analysis is restricted to average magnitudes observed in the set under study and not in the behaviors displayed by each of the agents in particular), in which agents update their strategies through imitation of the players with higher accumulated payoffs. The choice of roles (dictator vs. receiver) is assigned randomly, although with a probability that may be different from 50%. On the one hand, what makes^46^ particularly relevant is that the interaction between agents is mediated by a complex network structure, which serves to simulate that real populations have elaborate social structures; something that has not been considered in this work. On the other hand, another interesting aspect of Ref.^46^ is that it shows results very much aligned with experimental results (and therefore with the results of the present work) for the case where dictator-recipient roles are assigned with 50% probability, and with some independence of the underlying network structure as proposed in our work (although experimental results seem to be especially well captured by the model in Ref.^46^ when a free-scale network^48^ is used to stablish the relationship between). This could point to the fact that endowing agents with a complex social structure may not be necessary to understand the dynamics that lead to establishing the scaled adoption of cooperative behaviors, an insight which would be aligned with the results of this work. Further research could be needed in this regard, since the adoption of social structures could improve the representation of normative interactions in a work in which these are explicitly represented. In that vein, it could be thought that the updating of expectations in turn leads to changes in the social structure by homophily-type phenomena, so that reference groups with different norms could be constituted. This is something that goes beyond the scope of the present work, but that will be considered for future developments.

**Figure 7:**
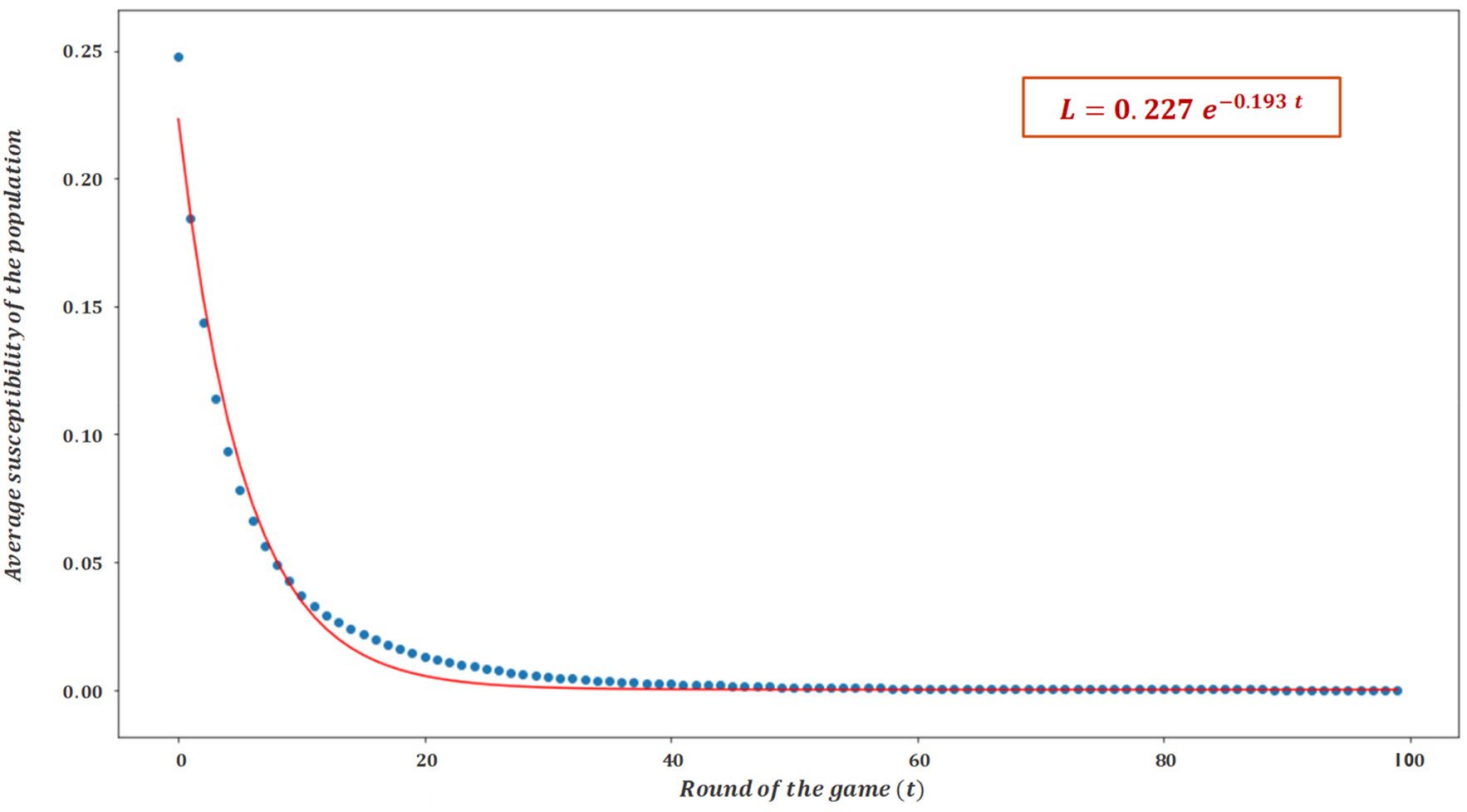
Evolution of agents’ average susceptibility for ${W}_{ }^{emp,neg}={W}_{ }^{nor,neg}=1$, ${W}_{ }^{emp,pos}={W}_{ }^{nor,pos}=\frac{1}{3}$ and ${l}_{i,t}^{nor}={l}_{i,t}^{emp}$.

Alternatively, Ref.^47^ is a work that studies the adaptation of agents to cooperative and non-cooperative environments (modeled by means of the coordination game^49^) through the generation and adjustment of empirical expectations based on the reinforcement learning mechanism. The reason it turns out to be a work of particular relevance, despite the fact that it does not study the interaction between agents through the DG, is that it shows that in order to match the model results with the experimental results, the learning parameter modulating the updating of expectations has to follow an exponential law, which is in excellent agreement with the results observed in Fig. 7. It is particularly noteworthy that the exponential law describing the learning presented in Ref.^47^ also shows strong similarities with that observed in the present work, since in addition to taking similar values (both learning parameters fall in the range [0; 0.25]), they decay with a similar time constant (both parameters reach their stationary value after about 20 rounds of play).

## Conclusions

In this paper we have proposed a model based on the mechanism of reinforcement learning to simulate the interaction dynamics of a set of agents through the Dictator Game (DG). Given that the results of the model mimic some key representative features of real-life experiments, the model itself might have considerable potential to capture the cognitive-behavioral processes that shape decision making in potentially collaborative environments through a candidate theory to explain collaborative conduct in situations similar to DG settings: the theory of social expectations.

By comparing the results arising from different possible formulations (see Annex), we have seen that the model describes the experiments better when agents are influenced in an analogous way by normative interactions and by empirical interactions, so the best way to capture the sensitivity to the stimuli arising from these interactions is through a single parameter. Furthermore, in a context where negative interactions are set to be more influential than positive interactions in shaping learning processes (in agreement with psychological literature), both empirical and normative interactions seem to be relevant to correctly capture the process of human strategic evolution, although the former are often dominant.

In the future, it would be interesting to represent the psychological profiles of different agents through a more detailed characterization of the impact of positive and negative interactions of an empirical and normative nature, for example, by endowing agents with personal normative beliefs, and making the evolution of agents' expectations also influenced by this parameter. In this vein, another stimulating line of research would be to further explore how positive and negative interactions can produce population-wide susceptibility phase-shifts, such as the ones observed in this paper. In addition, considering other network settings, different from the fully connected network approach assumed in this paper, would enable studying the role of the so-called "reference network" in normative interactions and, furthermore, would allow to account for other possibly relevant effects such as homophily and social expectation-network coevolution. Future work could also be targeted at developing a framework to assess how decision-makers address prosocial policy (e.g., subsidy policy), especially in scenarios subject to risk, where the endowment effect might play a relevant role.

## Supplementary Information

Supplementary Information.

## Data Availability

The datasets used and analysed during the current study available from the corresponding author on reasonable request.
