# Supplementary material for: A reinforcement learning approach to explore the role of social expectations in altruistic behavior
Source: Sci Rep. 2023 Jan 31;13:1717. doi: 10.1038/s41598-023-28659-0 (PMC9889354; doi:10.1038/s41598-023-28659-0)
Supplement: Supplementary file 1 — Supplementary Information. [file 41598_2023_28659_MOESM1_ESM.docx]

# Annex

In this annex, we present results on the more general case in which agents are allowed to update their susceptibilities in different ways when interacting normatively or empirically. This allows for agents who, for example, give a significant weight to interactions of an empirical nature $\left( l_{i,t}^{emp}\approx1 \right)$ and a negligible weight to those of a normative nature $\left( l_{i,t}^{nor}\approx0 \right)$, something that was not allowed in the formulation used for the case study presented in the main body of the paper. Analogously to the former case study, we present settings where two impact levels are kept static, while the other two vary in even 1/3 steps. In particular, we first show the case with empirical impact levels equal to zero $\left( W^{emp}=0 \right)$, followed by the parallel case where $W^{nor}=0$. Then, we show the results corresponding to the sets of larger impact levels ($W^{emp}=1$ and $W^{nor}=1$), and present the most relevant insights that can be derived from the joint analysis of all four figures.

Figure 1 Final averaged distributions of donations and susceptibility profiles for $W^{emp}=0$ and $l_{i,t}^{nor}\neq l_{i,t}^{emp}$. Each subplot represents results for a given combination of $W^{nor,pos} \left( which varies across rows \right)\mathrm{and}W^{nor,neg} \left( which varies across columns \right)$

Figure 2 Final averaged distributions of donations and susceptibility profiles for $W^{nor}=0$ and $l_{i,t}^{nor}\neq l_{i,t}^{emp}$. Each subplot represents results for a given combination of $W^{emp,pos} \left( which varies across rows \right)\mathrm{and}W^{nor,neg} \left( which varies across columns \right)$

Figure 3 Final averaged distributions of donations and susceptibility profiles for $W^{emp}=1$ and $l_{i,t}^{nor}\neq l_{i,t}^{emp}$. Each subplot represents results for a given combination of $W^{nor,pos} \left( which varies across rows \right)\mathrm{and}W^{nor,neg} \left( which varies across columns \right)$

Figure 4 Final averaged distributions of donations and susceptibility profiles for $W^{nor}=1$ and $l_{i,t}^{nor}\neq l_{i,t}^{emp}$. Each subplot represents results for a given combination of $W^{emp,pos}\left( which varies across rows \right) \mathrm{and}W^{emp,neg} \left( which varies across columns \right)$

Figures 2 and 3 the lower left corner subplots of Figure 1 and 9 (where $W^{nor}=W^{emp}=0$) show the system’s evolution in a case where susceptibility levels are forced to be static and equal to their original value (i.e., $l_{i,t}^{emp}=l_{i,t=0}^{emp} ; l_{i,t}^{nor}=l_{i,t=0}^{nor}$). Once again, the evolution rules leads to an equitable sharing solution in the stationary regime, as the average grant is located at 50% of the initial endowment. This seems to convey that the “fair” distribution may arise from multiple starting conditions, showing some robustness to the way initial conditions are set.

In a similar vein to the results presented in the case study shown in the main body of this work, Figures 8-11 also indicate that negative interactions tend to govern the interaction dynamics between agents. This is derived from observing that changes over $W^{emp,neg}$ and $W^{nor,neg}$, holding all other parameters constant, lead to transform the distributions of donations (and the evolution of susceptibilities) more significantly than changes in the parameters that account for positive interactions ($W^{emp,por}$ and $W^{nor,pos}$). As in the previous case, larger values of $W^{emp,neg}$ $W^{nor,neg}$ tend to provide average donations that are closer to the ones provided by Engels meta-analysis [19] (in the range of $\left[ \frac{3}{10}\Phi,\frac{4}{10}\Phi\right]$), and further away from the equitable distribution.

As a global conclusion from this case study, and as stated above, it seems that allowing socially “inconsistent” behavior (i.e., allowing agents to be reactive to normative interactions and not to empirical ones, or viceversa, something that arises from $l_{i,t}^{nor}\neq l_{i,t}^{emp}$) results in a large loss of heterogeneity in the final donation profiles, something that is in strong misalignment with experimental evidence.
